# Mitochondrial Haplotype Shapes the Trajectory of Ovarian Aging in Genetically Heterogeneous Rats

**DOI:** 10.1111/acel.70710

**Published:** 2026-09-10

**Authors:** Subhasri Biswas, Hoang V. M. Nguyen, Aubrey Converse, Satoshi Matsuzaki, Michael T. Kinter, Kenneth M. Humphries, Willard M. Freeman, Sarah R. Ocañas, Arlan Richardson, Francesca E. Duncan, Tommy L. Lewis, Michael B. Stout

**Affiliations:** ^1^ Aging and Metabolism Research Program Oklahoma Medical Research Foundation Oklahoma City Oklahoma USA; ^2^ Department of Biochemistry and Physiology, College of Medicine University of Oklahoma Health Sciences Oklahoma City Oklahoma USA; ^3^ Department of Obstetrics and Gynecology, Feinberg School of Medicine Northwestern University Chicago Illinois USA; ^4^ Genes & Human Disease Research Program Oklahoma Medical Research Foundation Oklahoma City Oklahoma USA; ^5^ Oklahoma City Veterans Affairs Medical Center Oklahoma City Oklahoma USA

**Keywords:** anti‐Müllerian hormone, follicle, haplogroup, menopause, oxidative phosphorylation, rat, reproductive senescence

## Abstract

Ovarian aging leads to permanent reproductive senescence and systemic hormonal changes that predispose women to age‐associated comorbidities. Despite these observations, the intrinsic mechanisms driving age‐related ovarian decline are poorly defined. Mitochondrial DNA (mtDNA) mutations and instability are strongly associated with aging; however, it remains unknown if naturally occurring mitochondrial genetic variation influences the trajectory of ovarian aging. To address this, we compared two genetically heterogeneous rat cohorts (OKC‐HET^B^ and OKC‐HET^W^) that differ in mitochondrial haplotype on a randomized but equivalently distributed nuclear background. The OKC‐HET^W^ haplotype was associated with accelerated loss of primordial follicles and pathological remodeling marked by fibrosis, macrophage infiltration, and multinucleated giant cells. These tissue‐level pathologies were paralleled by mitochondrial dysfunction, characterized by decreased respiratory complex activity, ATP production, and mtDNA copy number. Mechanistically, we identified a haplotype‐specific defect in mitochondrial genome maintenance. Although TFAM expression was normal, and total TFAM protein was elevated, OKC‐HET^W^ ovaries showed reduced mitochondrial TFAM abundance, TFAM‐mtDNA binding, and TOMM20, suggesting that impaired TOMM20‐mediated import is associated with compromised mitochondrial genomic stability. Longitudinal transcriptomic and proteomic analyses further indicate that mitochondrial haplotype influences the rate of ovarian aging, with OKC‐HET^W^ ovaries showing accelerated activation of inflammatory and fibrotic pathways alongside suppressed proteostasis and mitochondrial function. These defects corresponded to impairments in ovulation and a trend toward worsening oocyte quality. Collectively, our findings identify mitochondrial haplotype as a heritable modifier of ovarian aging rate that acts in concert with the nuclear genome, and a putative target for preserving ovarian function and female healthspan.

## Graphical abstract

Using genetically heterogeneous rats carrying divergent mitochondrial haplotypes on a randomized nuclear background, we show that mtDNA variation modifies the rate of ovarian aging. OKC‐HET^W^ ovaries showed bioenergetic deficits, reduced mtDNA copy number, and impaired TFAM–mtDNA binding by 4 months of age, with early fibrosis and immune infiltration, preceding accelerated loss of the primordial follicle reserve and reduced ovulatory capacity. Reduced TOMM20 abundance implicates impaired mitochondrial protein import, acting in concert with the nuclear genome.

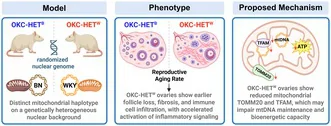

## 1 Introduction

The female reproductive system is one of the earliest organ systems to undergo aging in humans, with ovarian function declining decades before most other tissues (Duncan and Gerton 2018; Broekmans et al. 2009). Although ovaries are best known for their reproductive roles, they also function as endocrine organs regulating systemic physiology, as evidenced by the increased risks of osteoporosis, cardiovascular disease, and cognitive decline following oophorectomy or menopause (Hodis and Mack 2022; Nerattini et al. 2023; Stevenson and S. medical advisory council of the British Menopause 2023; te Velde et al. 1998; Wellons et al. 2012). Although life expectancy has increased in recent centuries, age at menopause has remained relatively stable (Duncan and Gerton 2018; Talaulikar 2022). Consequently, women spend an increasing proportion of their lives in the post‐reproductive state, underscoring ovarian aging as a key contributor to chronic disease burden and health outcomes. Ovarian aging is characterized by a gradual loss of the primordial follicle pool and declining oocyte quality, as well as extensive remodeling of the ovarian microenvironment with the emergence of multiple aging hallmarks within the ovary (Camaioni et al. 2022; Wu et al. 2022). These include mitochondrial dysfunction, oxidative stress, chronic inflammation, progressive stromal fibrosis, and the age‐dependent accumulation of multinucleated giant cells (MNGCs), a macrophage‐derived population that is abundant in aged ovaries and linked to chronic inflammation (Briley et al. 2016; Converse et al. 2025; Isola, Hense, et al. 2024; Isola, Ocañas, et al. 2024; Wu et al. 2025). Although the cellular and molecular mechanisms underlying these hallmarks are still being defined, their presence coincides with increased risk of oocyte aneuploidy, miscarriage, and adverse reproductive outcomes (Nagaoka et al. 2012; Nybo Andersen et al. 2000; Takahashi et al. 2011). Importantly, the temporal dynamics and magnitude of ovarian aging differ considerably among individuals, with some experiencing early depletion of the ovarian reserve and accelerated functional decline, while others maintain ovarian activity well into late life (te Velde and Pearson 2002). This inter‐individual variability suggests that intrinsic determinants, rather than chronological age alone, govern the rate and manner in which ovarian aging occurs.

Mitochondria are central players in ovarian physiology, as the high metabolic demands of both oocytes and ovarian somatic cells require robust mitochondrial function to sustain folliculogenesis, steroidogenesis, meiotic progression as well as early embryonic development (Babayev and Seli 2015; Chappel 2013). With advancing age, ovarian mitochondrial function progressively declines, marked by diminished oxidative phosphorylation efficiency, reduced ATP production, accumulation of mitochondrial DNA (mtDNA) damage, altered mitochondrial dynamics, and changes to the mitochondrial proteome, ultimately leading to impaired oocyte quality and reduced fertility (Bentov et al. 2011; May‐Panloup et al. 2016; Wang et al. 2017; Bomba‐Warczak et al. 2024). However, whether differences in mtDNA contribute to variability in ovarian aging trajectories has yet to be tested experimentally. This question is particularly relevant because human populations exhibit extensive genetic diversity in both the nuclear and mitochondrial genomes (Torroni et al. 1996; Wallace 1994). mtDNA is highly polymorphic, and specific combinations of maternally inherited single nucleotide polymorphisms define mtDNA haplogroups that indirectly reflect the evolution of human populations (MITOMAP: A Human Mitochondrial Genome Database 2026). Notably, mitochondrial haplogroup variation has been linked to measurable differences in mitochondrial bioenergetics, oxidative stress response, and susceptibility to a range of pathologies (Gomez‐Duran et al. 2010; Ruiz‐Pesini et al. 2000; Carrieri et al. 2001; Lorente et al. 2013; Saxena et al. 2006). Emerging human studies suggest that specific mitochondrial haplogroups may affect ovarian aging. May‐Panloup et al. (May‐Panloup et al. 2014) found that mtDNA haplogroup distribution differed in a cohort of 200 Caucasian women, when comparing women with diminished ovarian reserve with those having normal ovarian reserves. Importantly, carriers of the JT macro‐haplogroup had an approximately threefold lower risk of premature ovarian reserve depletion compared to women in other haplogroups. Likewise, Cheng et al. (Cheng et al. 2025) observed that Taiwanese women carrying haplogroup R had a reduced risk of showing diminished ovarian response. In this haplogroup, markers of cellular aging, including reactive oxygen species levels, mtDNA copy number, and telomere length, were age‐independent, and oocyte yield did not decline in contrast to other haplogroups, further supporting a possible protective effect of specific mtDNA lineages against reproductive aging. Even so, human studies are observational, and mitochondrial haplogroup is difficult to separate from nuclear ancestry or environmental factors. Thus, it remains unresolved whether mtDNA variation modulates ovarian aging, and through which mechanisms. Addressing this requires an experimental system in which mitochondrial haplotype is defined, environmental exposure is standardized, and nuclear genetic variation is randomized with respect to haplotype rather than confounded with it.

Interpreting such a comparison requires the recognition that mitochondrial function is not encoded solely by mtDNA. The mitochondrial proteome comprises more than a thousand nuclear‐encoded proteins alongside the thirteen polypeptides encoded by mtDNA, and expression of the two genomes must be coordinately regulated for oxidative phosphorylation to proceed (Quiros et al. 2016). Therefore, the phenotypic consequence of a given mitochondrial haplotype is expected to depend, at least in part, on the nuclear background against which it is expressed. Intraspecific mtDNA variation placed on a fixed nuclear background is sufficient to alter metabolism, insulin signaling, and healthspan in mice and rats (Houstek et al. 2012; Latorre‐Pellicer et al. 2016; Pravenec et al. 2007), and mitonuclear epistasis has been resolved to a nucleotide‐level interaction between a mitochondrially encoded tRNA and its nuclear‐encoded synthetase in *Drosophila*, in which each variant was benign on its own genetic background and deleterious in combination, reducing the activities of complexes I, III, and IV (Meiklejohn et al. 2013). Thus, the operative genetic unit in any comparison of haplotypes is the mitonuclear combination, rather than mtDNA in isolation.

In the present study, we utilized a genetically heterogeneous rat model generated through a 4‐way cross of inbred strains (Brown Norway [BN], Fischer 344 [F344], Lewis [LEW], Wistar Kyoto [WKY]). Reciprocal matings produced cohorts with distinct mitochondrial haplotypes, OKC‐HET^B^ (BN mtDNA) and OKC‐HET^W^ (WKY mtDNA), which differ at 94 nucleotides (Sathiaseelan et al. 2023), a divergence comparable to that separating human mitochondrial haplogroups (Merriwether et al. 1991). Because both cohorts descend from reciprocal crosses of the same two F1 lines, every animal inherits one recombinant BN/F344 and one recombinant WKY/LEW haploid autosomal genome, so the expected contribution of each founder strain is identical between cohorts while individual nuclear genotypes differ. Nuclear background is therefore randomized with respect to, rather than confounded with, mitochondrial haplotype. This model has previously been characterized across multiple organ systems, with mitochondrial haplotype influencing physical function and the metabolic response to 17α‐estradiol treatment (Sathiaseelan et al. 2023), the plasma metabolome, lipidome and proteome (Nguyen et al. 2026), gut microbiome composition and microbial metabolites (Nguyen et al. 2025), and adaptation to endurance exercise training (Ahn et al. 2025), with each report demonstrating trait‐ and sex‐dependent effects. This design enabled us to examine whether mitochondrial haplotype influences ovarian aging in the setting of a genetically diverse nuclear background. We found that mitochondrial haplotype was associated with differences in the trajectory of ovarian aging, with the OKC‐HET^W^ haplotype exhibiting accelerated primordial follicle pool depletion, early stromal fibrosis, enhanced inflammatory remodeling, and impaired ovulatory capacity. Mechanistically, these differences were associated with early defects in mitochondrial bioenergetic function and altered mitochondrial transcription factor A (TFAM)‐mediated mtDNA maintenance. To our knowledge, this is the first report to evaluate mitochondrial haplotype as a modifier of ovarian aging trajectory in a model with defined nuclear genetic diversity, in which mitochondrial genetic variation is assessed against a randomized, but equivalently distributed, nuclear background.

## 2 Results

### 2.1 Age‐Dependent Changes in Ovarian Reserve Differ by Mitochondrial Haplotype in OKC‐HET ^B/W^ Rats

**FIGURE 1:**
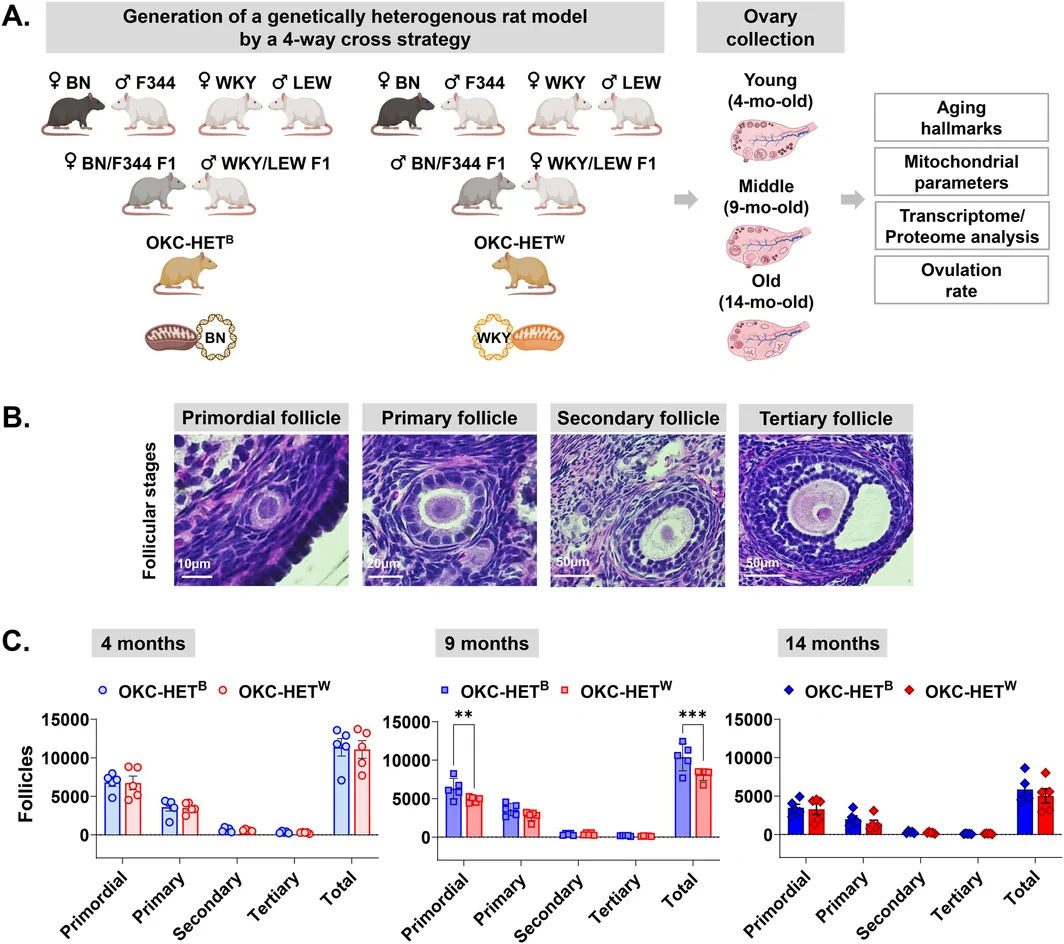
Mitochondrial haplotype influences the rate of decline in ovarian reserve. (A) Schematic of the four‐way cross strategy used to generate OKC‐HET^B^ and OKC‐HET^W^ rats with distinct mitochondrial haplotypes, (B) Representative H&E stained ovarian sections illustrating follicle classification criteria for primordial, primary, secondary, and tertiary follicles, and (C) Estimated numbers of follicles at each developmental stage in OKC‐HET^B^ and OKC‐HET^W^ female rats at 4, 9, and 14 months of age. All data are presented as mean ± SEM and analyzed by two‐way ANOVA with Tukey post hoc test. *n* = 5/haplotype/timepoint; **p < 0.01; ***p < 0.001.

To assess the role of mitochondrial haplotype on ovarian aging, we used a rat model developed through a four‐way cross of commercially available inbred strains, producing two F2 lines with diverse nuclear genomes which differ by mitochondrial haplotype (OKC‐HET^B^ or OKC‐HET^W^). At 4, 9, and 14 months of age, ovaries were collected to capture reproductively young, reproductively middle‐aged, and reproductively old stages, respectively, for downstream histological and quantitative analyses (Figure 1A). Follicles were classified as primordial, primary, secondary, and tertiary using established histological criteria (Figure 1B). Quantitative follicle counting revealed an age‐dependent decline in follicle populations in both haplotypes with advancing age (Figure 1C), but the timing and magnitude of follicle loss differed in a mitochondrial haplotype‐dependent manner. At 4 months of age, follicle numbers at all stages were comparable between OKC‐HET^B^ and OKC‐HET^W^ rats. Conversely, by 9 months of age, both the primordial follicle pool and total follicle count were significantly reduced in OKC‐HET^W^ ovaries when compared to age‐matched OKC‐HET^B^ rats, indicating an accelerated depletion of the ovarian reserve. By 14 months of age, follicle numbers had declined substantially in both haplotypes and no longer differed by haplotype at any stage. These results indicate that mitochondrial haplotype is associated with the rate at which the ovarian reserve is depleted, rather than with its initial establishment, with the OKC‐HET^W^ haplotype displaying an accelerated exhaustion of the primordial follicle pool.

### 2.2 Age‐Dependent Ovarian Fibrosis and MNGC Accumulation Differ by Mitochondrial Haplotype in OKC‐HET ^B/W^ Rats

**FIGURE 2:**
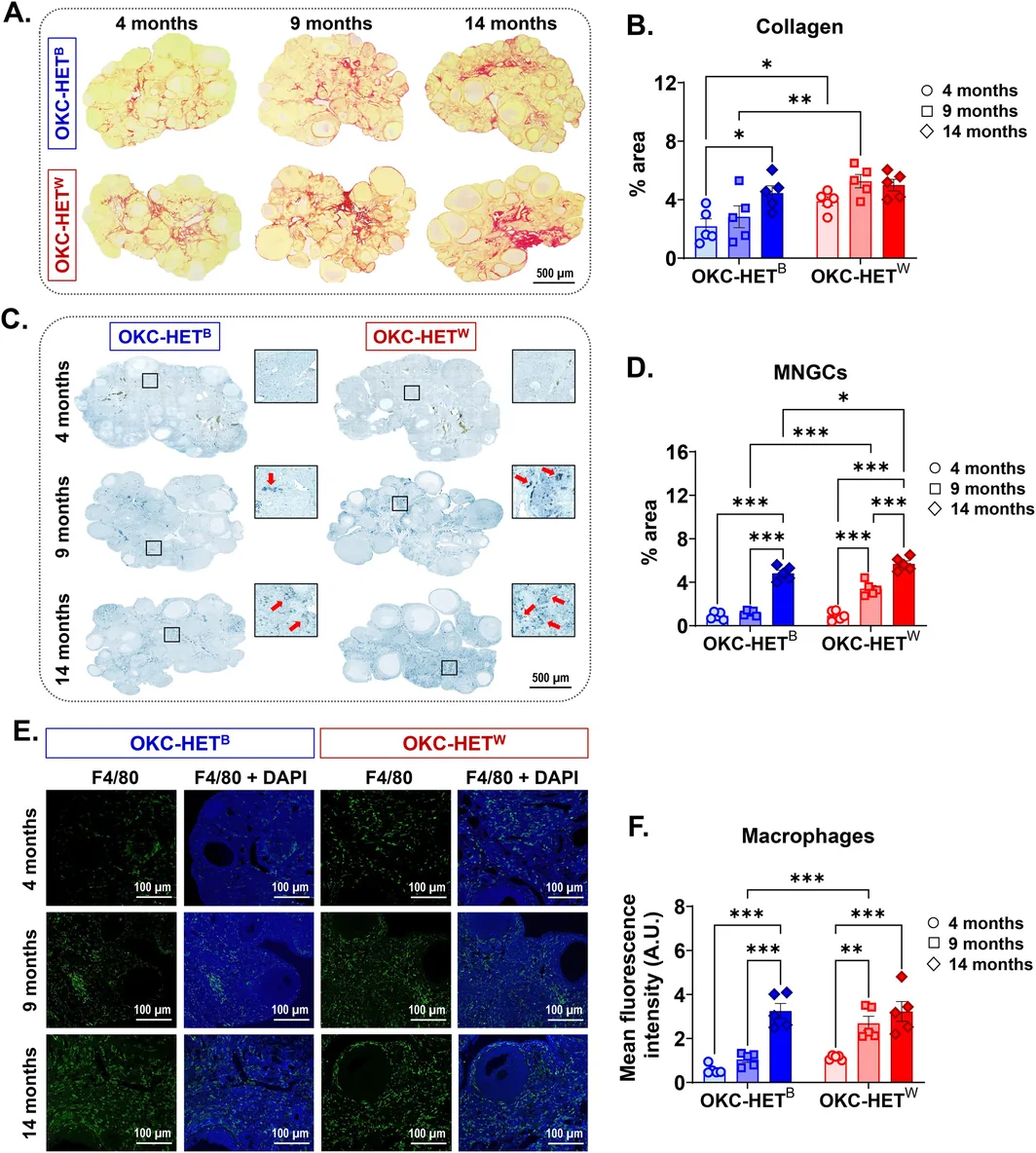
Mitochondrial haplotype influences the emergence of ovarian fibrosis, MNGC formation, and macrophage accumulation. (A) Representative Picrosirius Red stained ovarian sections, (B) Quantification of ovarian collagen abundance expressed as the percentage of Picrosirius Red‐positive area relative to the total ovarian tissue area, (C) Representative Sudan Black B stained ovarian sections, (D) Quantification of ovarian MNGC abundance expressed as the percentage of Sudan Black B‐positive area relative to the total ovarian tissue area, (E) Representative immunofluorescence images of ovarian F4/80 positivity (green) and nuclei (blue), and (F) Quantification of ovarian macrophage abundance expressed as mean F4/80 fluorescence intensity measured within regions of interest of constant area from OKC‐HET^B^ and OKC‐HET^W^ at 4, 9, and 14 months of age. All data are presented as mean ± SEM and analyzed by two‐way ANOVA with Tukey post hoc test. *n* = 5/haplotype/timepoint/assay; *p < 0.05; **p < 0.01; ***p < 0.001.

To investigate how mitochondrial haplotype influences age‐related alterations in the ovarian microenvironment, we assessed fibrosis and MNGC emergence across ages. Picrosirius Red staining showed that the age‐related increase in collagen deposition was progressive in OKC‐HET^B^ ovaries (Figure 2A,B). In contrast, OKC‐HET^W^ ovaries displayed a pronounced increase in fibrosis at 4 months of age when compared to OKC‐HET^B^ ovaries, and these levels remained elevated at 9 and 14 months of age (Figure 2A,B). Given that ovarian fibrosis is closely linked to inflammation, we next examined MNGCs, a macrophage‐derived population that accumulates in the ovary with advancing age. Sudan Black B staining identified lipofuscin‐rich MNGCs within the ovarian stroma (Figure 2C). While MNGCs were rare in young ovaries from both haplotypes, their abundance increased sharply at 9 and 14 months of age in OKC‐HET^W^ ovaries when compared with age‐matched OKC‐HET^B^ ovaries (Figure 2D). Immunofluorescence analysis further confirmed that both haplotypes had an age‐related increase in ovarian macrophages (F4/80^+^). Macrophage abundance did not differ between haplotypes at 4 months of age but was significantly greater in OKC‐HET^W^ ovaries by 9 months, with both haplotypes reaching comparable levels by 14 months (Figure 2E,F). In contrast, total leukocyte abundance assessed by CD45 immunofluorescence was already significantly greater in OKC‐HET^W^ ovaries at 4 months of age (Figure S1A,B), indicating that the shift in ovarian immune composition precedes expansion of the macrophage compartment. Together, these data suggest that the earlier emergence of fibrosis within OKC‐HET^W^ ovaries is associated with an earlier shift in immune cell composition, subsequent macrophage accumulation, and MNGC formation, supporting the notion that mitochondrial haplotype modulates fibrotic and immune remodeling during the ovarian aging process.

### 2.3 Mitochondrial Haplotype Influences Ovarian Mitochondrial Bioenergetics and mtDNA Regulation in OKC‐HET ^B/W^ Rats

**FIGURE 3:**
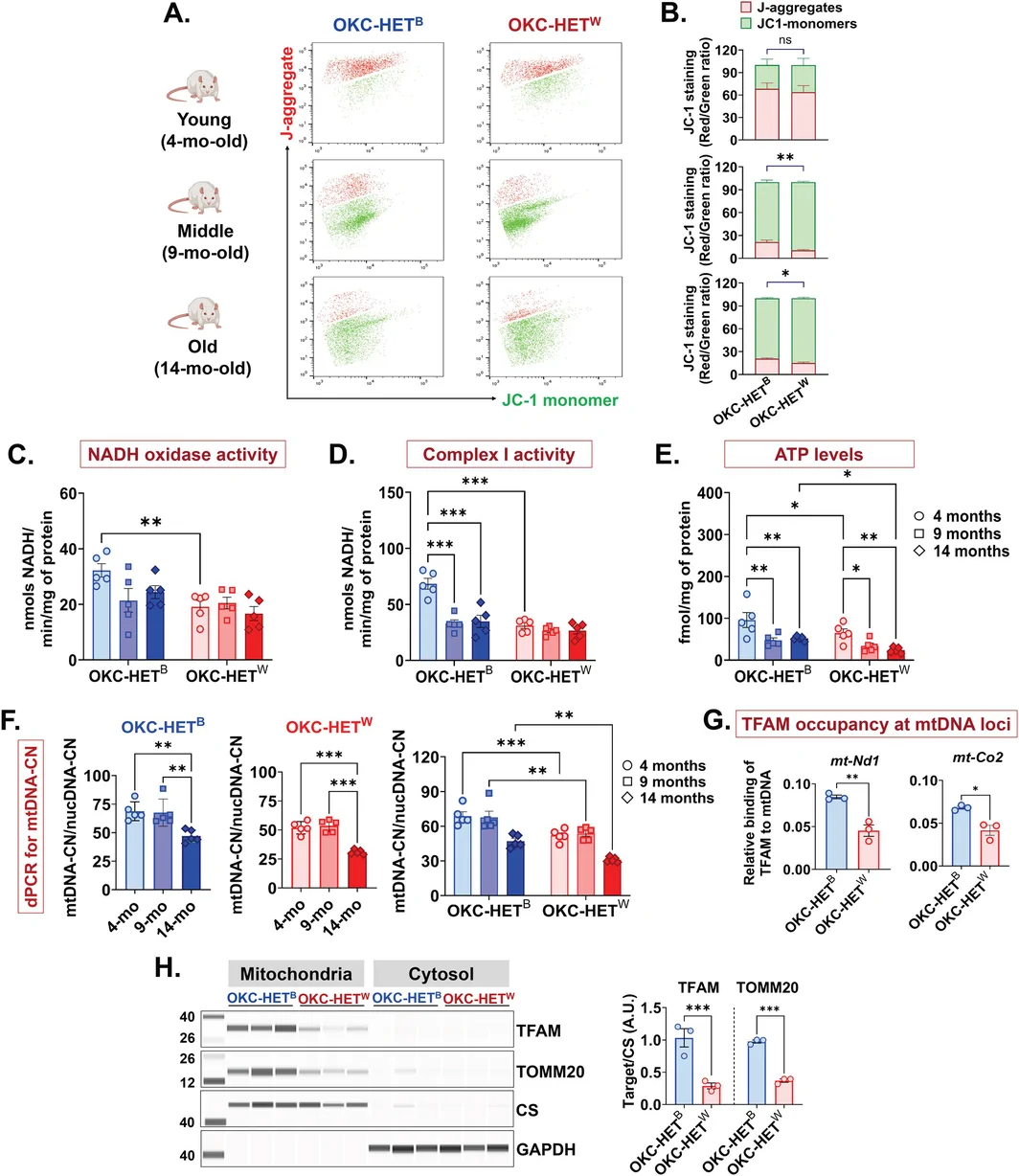
Mitochondrial haplotype influences mitochondrial energetic remodeling in the aging ovary. (A) Representative bivariate flow cytometry plots depicting JC‐1 aggregate (red; polarized mitochondria) and JC‐1 monomer (green; depolarized mitochondria) fluorescence in single‐cell ovarian suspensions, (B) Quantification of JC‐1 aggregate and monomer fluorescence expressed as the JC‐1 aggregate‐to‐monomer (red/green) fluorescence ratio, (C‐D) Enzymatic activities of ovarian mitochondrial NADH oxidase and respiratory chain complex I, (E) Ovarian ATP levels, and (F) Ovarian mitochondrial DNA (mtDNA) copy number measured by digital PCR and normalized to nuclear genomic DNA from OKC‐HET^B^ and OKC‐HET^W^ at 4, 9, and 14 months of age, (G) ChIP‐dPCR analysis of TFAM occupancy at the *mt‐Nd1* and *mt‐Co2* loci, and (H) Representative immunoblots of TFAM, TOMM20, citrate synthase (CS), and GAPDH in mitochondrial and cytoplasmic fractions, with quantification of mitochondrial TFAM and TOMM20 protein abundance normalized to citrate synthase (CS) in ovaries from OKC‐HET^B^ and OKC‐HET^W^ rats at 4 months of age. All data are presented as mean ± SEM and analyzed using *t*‐tests (panel A‐B; G‐H), one‐way ANOVA, or two‐way ANOVA with Tukey post hoc testing (panel C–F), as appropriate. *n* = 3–5/haplotype/timepoint/assay; *p < 0.05; **p < 0.01; ***p < 0.001.

Given the haplotype‐dependent differences in ovarian aging phenotypes between OKC‐HET^B^ and OKC‐HET^W^, we next assessed whether these changes were associated with altered mitochondrial bioenergetics. To evaluate mitochondrial membrane potential, whole ovarian single cell suspensions were stained with JC‐1, which differentiates between polarized (red fluorescence; high mitochondrial activity) and depolarized (green fluorescence; low mitochondrial activity) mitochondria. As shown in Figure 3A,B, both OKC‐HET^B^ and OKC‐HET^W^ ovaries showed a similar J‐aggregate signal at 4 months of age. By 9 months of age, the J‐aggregate signal (red) declined in both haplotypes, reflecting an age‐related reduction in membrane potential, but the decline was greater in ovaries from OKC‐HET^W^ rats, which persisted at 14 months. We then quantified mitochondrial respiratory enzyme activities and ATP levels to further evaluate mitochondrial bioenergetic function. Both NADH oxidase and Complex I activities were significantly lower in ovaries from OKC‐HET^W^ rats by 4 months of age, whereas ovaries from OKC‐HET^B^ rats exhibited an age‐dependent decline, resulting in comparable activities by 9 months (Figure 3C,D). This observation suggests that mitochondrial respiratory defects occur prior to declines in membrane potential. Consistent with these findings, ATP levels in OKC‐HET^W^ ovaries were also significantly lower at both 4 and 14 months of age when compared to OKC‐HET^B^ (Figure 3E).

Since mitochondrial bioenergetic function is closely related to mitochondrial genome regulation, we then quantified mtDNA copy number in both haplotypes across reproductive ages. Both haplotypes displayed decreasing mtDNA copy numbers from 9 to 14 months of age, but OKC‐HET^W^ ovaries were consistently found to have lower mtDNA copy numbers than OKC‐HET^B^ at all ages analyzed (Figure 3F). Because mitochondrial transcription factor A (TFAM) plays a critical role in mtDNA packaging and stabilization, we next investigated whether the differences in mtDNA copy numbers between haplotypes were associated with differences in TFAM–mtDNA interactions. We employed chromatin immunoprecipitation (ChIP) assays directed against mtDNA‐encoded genes, *mt‐Nd1* and *mt‐Co2*, and found significantly decreased TFAM binding in ovaries from OKC‐HET^W^ compared to OKC‐HET^B^ rats at 4 months of age (Figure 3G). TFAM occupancy at the D‐loop, the non‐coding regulatory region containing the mitochondrial promoters and the origin of heavy‐strand replication, was not assessed. To determine if decreased TFAM–mtDNA binding correlated with altered TFAM expression, we next assessed TFAM transcript and protein levels. TFAM transcripts were comparable between OKC‐HET^B^ and OKC‐HET^W^ ovaries (Figure S2A), whereas total TFAM protein levels in whole ovarian lysates were significantly higher in OKC‐HET^W^ ovaries (Figure S2B), despite reduced TFAM–mtDNA binding. To determine whether the increased total TFAM protein reflected altered mitochondrial localization, we next performed subcellular fractionation of 4‐month‐old ovaries followed by immunoblotting analyses. TFAM abundance in the mitochondrial fraction was significantly reduced in OKC‐HET^W^ ovaries compared with OKC‐HET^B^ ovaries, whereas TFAM was not reliably detected in the cytoplasmic fraction under our experimental conditions (Figure 3H). To explore whether the reduced mitochondrial TFAM abundance occurred in the context of changes in mitochondrial protein import machinery, we next examined the expression of the mitochondrial outer membrane import receptor TOMM20. Blots from the same mitochondrial fractions demonstrated reduced TOMM20 abundance in OKC‐HET^W^ ovaries (Figure 3H). In contrast, abundance of the matrix‐localized TCA cycle enzyme citrate synthase (CS), which served as the mitochondrial loading reference for these analyses, was comparable between haplotypes (Figure 3H). The reductions in mitochondrial TFAM and TOMM20 were therefore selective, rather than reflecting a generalized decrease in mitochondrial protein abundance. Consistent with the reduction in mitochondrial TOMM20, immunofluorescence analysis of ovarian sections showed significantly reduced TOMM20 abundance in OKC‐HET^W^ ovaries relative to OKC‐HET^B^ at 4, 9, and 14 months of age (Figure S2C,D). These findings suggest that the import machinery for mitochondrial proteins is altered in OKC‐HET^W^ rats, which may contribute to the reduced TFAM–mtDNA binding observed in young ovaries. We note that the two haplotypes also differ in sequence within the D‐loop (Sathiaseelan et al. 2023), the regulatory region to which TFAM binds (Nguyen et al. 2026), which could independently influence TFAM–mtDNA association. These possibilities are not mutually exclusive and are considered further below.

Because reduced respiratory enzyme activity, ATP content, and mtDNA copy number could reflect a lower mitochondrial content rather than impaired mitochondrial function, we also quantified mitochondrial mass by MitoTracker staining of single‐cell ovarian suspensions. Mitochondrial mass declined between 4 and 9 months of age in both haplotypes and increased again by 14 months, with no differences between OKC‐HET^W^ and OKC‐HET^B^ ovaries at any age (Figure S3A). We additionally assessed regulators of mitochondrial dynamics. Abundance of the fission protein FIS1 increased with age in both haplotypes, whereas the fusion mediator MFN1 was unchanged across ages; neither differed between haplotypes at any age (Figure S3B,C). These findings indicate that the bioenergetic and mtDNA differences observed between haplotypes occur in the absence of a corresponding difference in mitochondrial content.

### 2.4 Age‐Dependent Ovarian Proteomic Remodeling Differs by Mitochondrial Haplotypes in OKC‐HET ^B/W^ Rats

**FIGURE 4:**
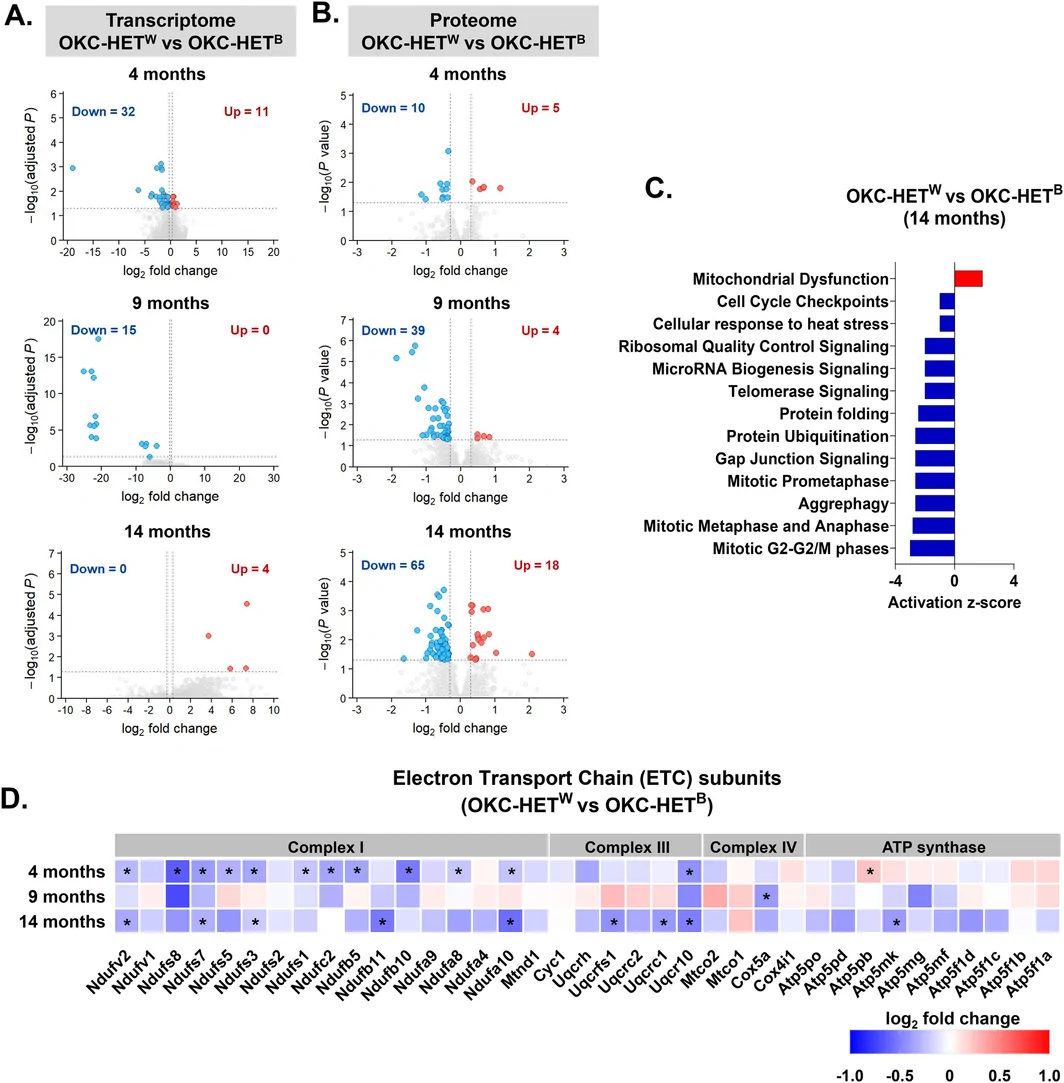
Across‐haplotype comparison reveals that mitochondrial haplotype influences the ovarian proteome in a time‐dependent manner. (A) Volcano plots showing differentially expressed transcripts between OKC‐HET^W^ and OKC‐HET^B^ ovaries following ovarian bulk RNA sequencing, (B) Volcano plots showing differentially expressed proteins between OKC‐HET^W^ and OKC‐HET^B^ ovaries following ovarian proteomic analyses by data‐independent acquisition, (C) IPA canonical pathway analysis of differentially expressed proteins between OKC‐HET^W^ and OKC‐HET^B^ ovaries at 14 months of age, and (D) Heatmap showing the relative abundance of ovarian mitochondrial respiratory chain (MRC) subunits identified within the data‐independent acquisition proteomic dataset from OKC‐HET^B^ and OKC‐HET^W^ at 4, 9, and 14 months of age. Differential transcript expression was assessed using DESeq2 (Wald test), and differential protein abundance was assessed using two‐way ANOVA with haplotype and age as factors, followed by Tukey post hoc testing for within‐age haplotype comparisons. *n* = 5/haplotype/timepoint/assay; transcripts with a false discovery rate < 0.05 and |log_2_FC| > 0.3, and proteins with *p* < 0.05 and |log_2_FC| > 0.3, were considered significant.

To establish whether the mitochondrial alterations identified between OKC‐HET^B^ and OKC‐HET^W^ ovaries were associated with global molecular events, we performed bulk transcriptomic and proteomic profiling at 4, 9, and 14 months of age. As shown in Figure 4A, the OKC‐HET^W^ ovaries showed 43 (11 upregulated, 32 downregulated), 15 (0 upregulated, 15 downregulated), and 4 (4 upregulated, 0 downregulated) differentially expressed genes at 4, 9, and 14 months, respectively, when compared to age‐matched OKC‐HET^B^ ovaries. The corresponding proteomic comparisons identified 15 (5 upregulated, 10 downregulated) and 43 (4 upregulated, 39 downregulated) differentially abundant proteins at 4 and 9 months of age, increasing to 83 (18 upregulated, 65 downregulated) by 14 months of age (Figure 4B). Pathway analysis of these proteins showed increased mitochondrial dysfunction and coordinated downregulation of pathways involved in cell cycle progression, protein homeostasis, and cellular stress response in OKC‐HET^W^ ovaries (Figure 4C), suggesting that mitochondrial haplotype‐dependent proteomic remodeling manifests more prominently as reproductive aging progresses. Considering the pathway enrichment of mitochondrial dysfunction in the proteomic analysis, we next examined the subset of the proteomic dataset corresponding to mitochondrial respiratory chain (MRC) subunits. This analysis revealed suppression of several OXPHOS subunits in OKC‐HET^W^ versus OKC‐HET^B^ ovaries, primarily driven by decreased abundance of complex I and III subunits (Figure 4D). Notably, all differentially abundant subunits were nuclear‐encoded. The three mtDNA‐encoded subunits detected in our dataset, Mtnd1, Mtco1, and Mtco2, did not differ between haplotypes at any age, indicating that these differences reflect altered abundance of nuclear‐encoded MRC components rather than of the mtDNA‐encoded subunits themselves. The remaining mtDNA‐encoded subunits were not detected, consistent with the limited recovery of small hydrophobic membrane proteins by bottom‐up proteomics. Interestingly, several subunits of the ETC that were different at 4 months of age were no longer significantly different at 9 months of age, but the differences reemerged at 14 months of age (Figure 4D). This is consistent with a compensatory response in reproductive middle age that temporarily buffers early life mitochondrial defects before more pronounced mitochondrial proteomic remodeling emerges later in reproductive life.

### 2.5 Mitochondrial Haplotype Influences Ovarian Aging Trajectories in OKC‐HET ^B/W^ Rats

**FIGURE 5:**
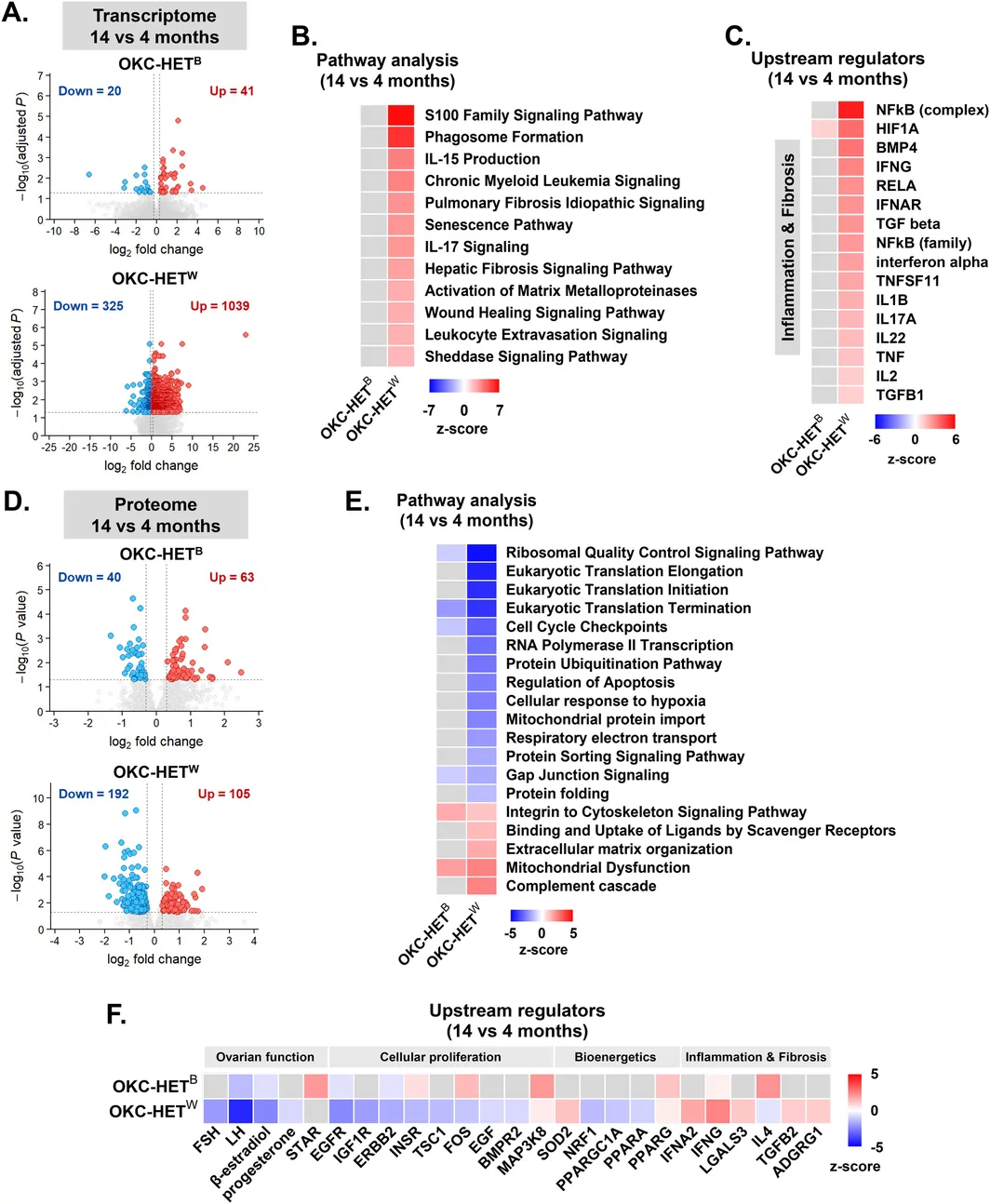
Within haplotype comparisons reveal distinct ovarian aging trajectories between mitochondrial haplotypes. (A) Volcano plots showing differentially expressed transcripts, (B) Heatmap of IPA canonical pathways, (C) Heatmap of IPA upstream regulators identified from transcriptomic comparisons within each haplotype following ovarian bulk RNA sequencing, (D) Volcano plots showing differentially expressed proteins, (E) Heatmap of IPA canonical pathways, and (F) Heatmap of IPA upstream regulators identified from proteomic comparisons within each haplotype using ovarian proteomic analysis by data‐independent acquisition, comparing 14‐ to 4‐month‐old ovaries. Differential transcript expression was assessed using DESeq2 (Wald test), and differential protein abundance was assessed using two‐way ANOVA with haplotype and age as factors, followed by Tukey post hoc testing for within‐haplotype age comparisons. *n* = 5/haplotype/timepoint/assay; transcripts with a false discovery rate < 0.05 and |log_2_FC| > 0.3, and proteins with *p* < 0.05 and |log_2_FC| > 0.3, were considered significant.

Next, to assess how ovarian aging diverges between mitochondrial haplotypes over time, we compared ovarian gene and protein expression in the ovaries of OKC‐HET^B^ and OKC‐HET^W^ rats at 14 versus 4 months of age within each haplotype. Transcriptomic analyses showed that age‐dependent changes in gene expression were markedly more pronounced in OKC‐HET^W^ (1039 upregulated, 325 downregulated) than OKC‐HET^B^ (41 upregulated, 20 downregulated) rats (Figure 5A). Pathway and upstream regulator analyses consistently demonstrated strong activation of inflammation and fibrosis‐associated signaling in OKC‐HET^W^ ovaries by 14 months of age relative to 4 months of age (Figure 5B,C). A similar divergence was evident at the level of the proteome, with 297 (105 upregulated, 192 downregulated) and 103 (63 upregulated, 40 downregulated) differentially abundant proteins in OKC‐HET^W^ and OKC‐HET^B^ ovaries, respectively (Figure 5D). Pathway analysis showed coordinated suppression of protein synthesis and proteostasis pathways in aged OKC‐HET^W^ ovaries, including ribosomal quality control, translation, transcription, protein folding, and ubiquitination, together with downregulation of cell‐cycle checkpoint signaling and respiratory electron transport (Figure 5E). Notably, mitochondrial protein import was among the pathways suppressed with age in OKC‐HET^W^ ovaries but not in OKC‐HET^B^ ovaries, providing independent support for the import deficit indicated by the reduced TOMM20 and mitochondrial TFAM abundance described above. Upstream regulator analysis of the proteomic data further revealed inhibition of regulators associated with ovarian function, cell proliferation, and mitochondrial function, along with activation of regulators controlling inflammation and fibrosis (Figure 5F). These molecular signatures parallel the age‐related histological features observed in OKC‐HET^W^ ovaries, including increased collagen deposition, MNGC accumulation, and macrophage infiltration. Together, they suggest that while the molecular differences between haplotypes in single time‐point comparisons are relatively modest, mitochondrial haplotype shapes how the ovary ages.

### 2.6 Ovulatory Capacity and Oocyte Quality Differ by Mitochondrial Haplotype in OKC‐HET ^B/W^ Females

**FIGURE 6:**
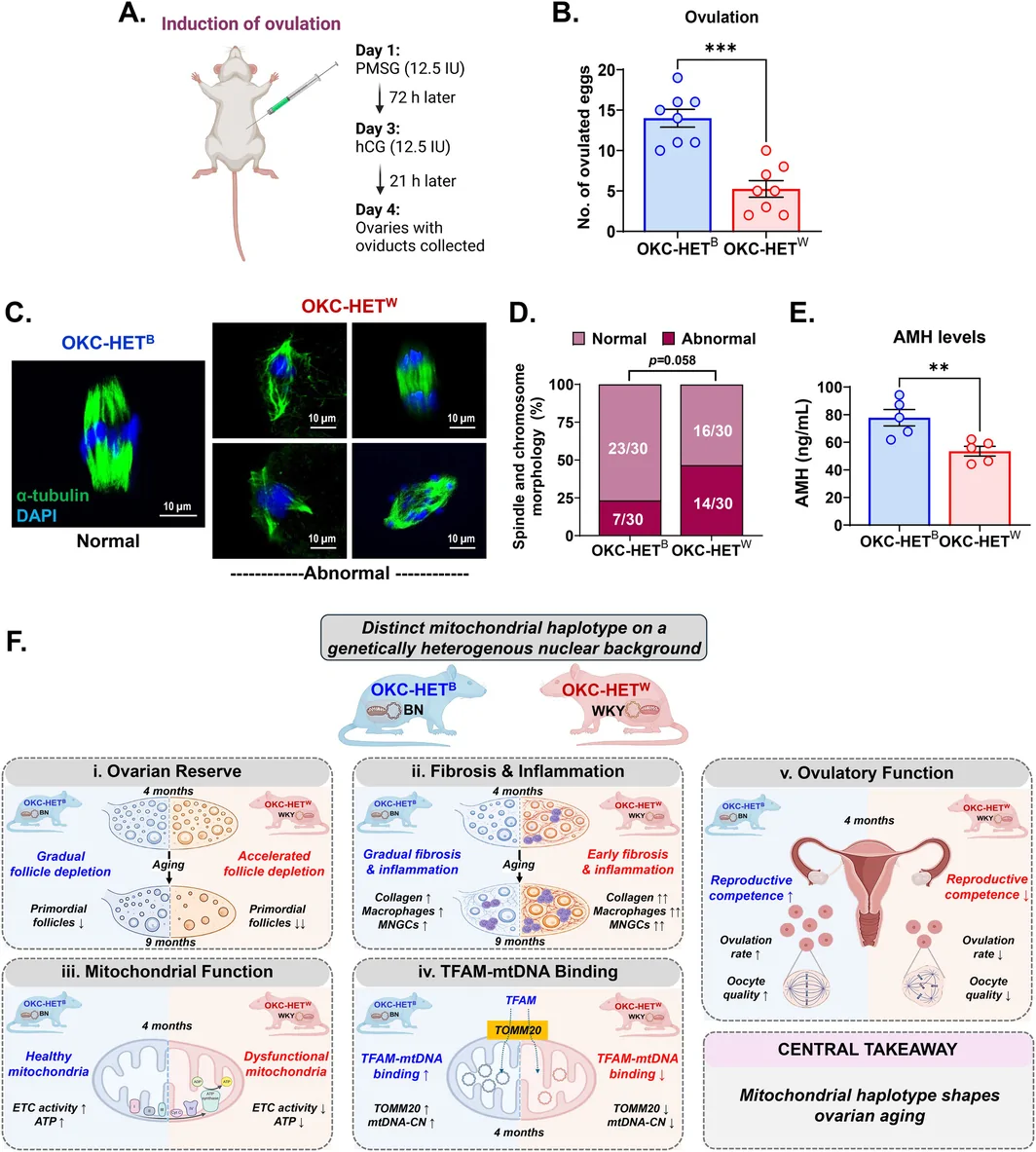
Mitochondrial haplotype influences ovulation efficiency and oocyte spindle organization. (A) Schematic illustrating hormonal induction of ovulation followed by assessment of ovulatory output and oocyte quality, (B) Quantification of the number of ovulated oocytes recovered per female following superovulation, (C) Representative confocal images of metaphase II oocytes stained for microtubules (α‐tubulin, green) and DNA (DAPI, blue) showing meiotic spindle morphology and chromosome alignment, (D) Quantification of normal and abnormal spindle morphology in metaphase II oocytes (30 oocytes per haplotype, randomly assessed from 8 females per haplotype), (E) Plasma anti‐Müllerian hormone (AMH) levels from female OKC‐HET^B^ and OKC‐HET^W^ rats at 4 months of age, and (F) A schematic summary showing that mitochondrial haplotype shapes distinct ovarian aging trajectories, with OKC‐HETᴮ females exhibiting gradual follicle depletion, preserved mitochondrial function, and maintained ovulatory competence, whereas OKC‐HETᵂ females show accelerated follicle loss, increased fibrosis, macrophage/MNGC accumulation, mitochondrial dysfunction, and reduced reproductive performance. Data in panels B and E are presented as mean ± SEM and analyzed using unpaired two‐tailed *t*‐tests; data in panel D are presented as proportions and analyzed using Chi‐square test. *n* = 8/haplotype for panels B and D; *n* = 5/haplotype for panel E; **p < 0.01; ***p < 0.001.

To investigate whether the mitochondrial and molecular defects identified in OKC‐HET^W^ ovaries are associated with impaired reproductive competence, we evaluated ovulation efficiency and oocyte spindle organization after hormonal stimulation of ovulation at 4 months of age (Figure 6A). As shown in Figure 6B, OKC‐HET^B^ females ovulated significantly more oocytes than OKC‐HET^W^ females, indicating impaired ovulatory capacity in the OKC‐HET^W^ haplotype. Meiotic spindle integrity and chromosome alignment were also evaluated as additional measures of oocyte quality. While OKC‐HET^B^ oocytes mostly displayed normal spindle formation, OKC‐HET^W^ oocytes were found to frequently exhibit abnormal spindle formations such as elongated or disorganized spindles, misaligned chromosomes, and multiple spindle structures (Figure 6C). Quantitative analysis of 30 oocytes per haplotype, recovered from 8 females per haplotype, identified abnormal spindle morphology in 14 of 30 (46.7%) OKC‐HET^W^ oocytes as compared with 7 of 30 (23.3%) OKC‐HET^B^ oocytes (*χ* ^2^ = 3.59, *p* = 0.058; Figure 6D). Plasma AMH levels were also found to be significantly lower in OKC‐HET^W^ females relative to OKC‐HET^B^ at 4 months (Figure 6E), suggesting follicle maturation may be lower in ovaries from OKC‐HET^W^ rats. Together, these findings indicate that mitochondrial haplotype influences ovarian aging across several biological scales ranging from mitochondrial bioenergetics and molecular remodeling to tissue pathology and ultimately oocyte quality and ovulatory capacity (Figure 6F).

## 3 Discussion

Ovarian aging shows considerable variability between individuals (te Velde and Pearson 2002) that cannot be fully accounted for by chronological age, indicating that intrinsic factors play a major role in shaping reproductive lifespan. Here we present experimental evidence that mitochondrial genetic variation influences the trajectory of ovarian aging. To address this, we employed a genetically heterogeneous rat model in which two mitochondrial haplotypes (OKC‐HET^B^ and OKC‐HET^W^) were compared against a randomized, but equivalently distributed, nuclear background (Sathiaseelan et al. 2023). Because haplotype is assigned by breeding design rather than co‐inherited with nuclear ancestry or lifestyle, this approach addresses a major limitation of prior human studies that described associations between mitochondrial haplogroups and ovarian reserve but could not separate mitochondrial effects from nuclear ancestry or environmental exposure (May‐Panloup et al. 2014; Cheng et al. 2025). Our data reveal that, across a diverse nuclear background, the OKC‐HET^W^ haplotype is associated with accelerated depletion of the primordial follicle reserve and premature remodeling of the ovarian microenvironment. To our knowledge, this is the first experimental evidence that mitochondrial genetic variation contributes to inter‐individual variation in the rate of ovarian aging.

Our data demonstrate that mitochondrial haplotype impacts the depletion dynamics of ovarian reserve but not its initial establishment or ultimate exhaustion. At 4 months of age (reproductively young), the numbers of follicles present in OKC‐HET^B^ and OKC‐HET^W^ ovaries were found to be comparable, as was the level of depletion at 14 months (reproductively old). However, at 9 months of age (reproductively middle‐aged), a marked reduction in the primordial follicle pool was evident in OKC‐HET^W^ ovaries, indicating an accelerated rate of depletion. An accelerated follicle pool depletion results in a compressed reproductive window and earlier endocrine and menopausal transitions in women (Broekmans et al. 2007; Faddy et al. 1992; Gold 2011). This not only increases the risk for early‐onset diminished ovarian reserve (DOR) and premature ovarian insufficiency (POI) (Cooper and Sandler 1998; De Vos et al. 2010), but also has broader implications for long‐term systemic health, as menopause is linked to increased risks of osteoporosis, cardiovascular disease, and cognitive decline (Hodis and Mack 2022; Nerattini et al. 2023; Stevenson and S. medical advisory council of the British Menopause 2023; te Velde et al. 1998; Wellons et al. 2012). These implications position mitochondrial haplotype as a heritable modifier of reproductive aging that acts in concert with the nuclear genome.

Intriguingly, we observed that the OKC‐HET^W^ haplotype promotes an aberrant ovarian microenvironment well before significant follicle loss occurs. Ovarian fibrosis was already elevated in OKC‐HET^W^ rats at 4 months of age and remained elevated at 9 and 14 months, whereas OKC‐HET^B^ ovaries exhibited a more gradual age‐dependent accumulation. This early fibrotic remodeling was coupled with immune infiltration. Total leukocyte abundance was already elevated in OKC‐HET^W^ ovaries at 4 months of age, whereas the macrophage‐specific increase emerged later, becoming significant by 9 months of age. This was followed by a marked and significant accumulation of lipofuscin‐rich MNGCs at later age (9 and 14 months) compared with OKC‐HET^B^ rats. MNGCs have been previously reported as a macrophage‐derived population that accumulates in the aged ovarian stroma in response to persistent accumulation of cellular debris resulting from cumulative ovulation, follicular atresia, and abnormal extracellular matrix deposition (Briley et al. 2016; Converse et al. 2025; Isola, Ocañas, et al. 2024; Foley et al. 2021), suggestive of a temporal sequence whereby fibrosis precedes MNGC formation. We speculate that this early fibrosis and inflammaging lead to the reported functional deficits as well. Though the follicle count at 4 months did not differ between haplotypes, OKC‐HET^W^ females had a significantly lower ovulation rate and a higher proportion of oocytes displaying abnormal meiotic spindles, although this observation did not reach statistical significance. This is in line with previous observations that stromal fibrosis impedes ovulation, and that antifibrosis agents (e.g., pirfenidone and BGP‐15) can reduce collagen deposition and restore fertility in reproductively old mice (Umehara et al. 2022; Lin et al. 2026). Together, these findings indicate that variability in the mitochondrial genome can influence the fibrotic and inflammatory remodeling, as well as reproductive potential, independent of age.

A growing body of evidence has recognized mitochondrial dysfunction and impaired bioenergetics as potential drivers of cellular stress, chronic inflammation, and fibrotic remodeling (Umehara et al. 2022; Zhao et al. 2024). In agreement with this, we observed haplotype‐specific defects in mitochondrial bioenergetics and genome maintenance. While the histological features of inflammaging become most apparent by reproductive middle age, our data indicate that these changes are preceded by functional mitochondrial deficits. Specifically, OKC‐HET^W^ ovaries exhibited reduced Complex I activity and lower ATP levels as early as 4 months of age. These findings suggest that early bioenergetic declines may create a microenvironment more permissive to inflammatory and fibrotic remodeling. Central to this possibility was the apparent disruption of mtDNA regulation. The inverse relationship between total TFAM protein abundance and TFAM–mtDNA binding suggests a failure of mitochondrial localization rather than of nuclear expression, a conclusion supported by the reduced TFAM abundance in the mitochondrial fraction. We surmise that this paradox may be explained by the accompanying reduction in TOMM20. As an integral part of the mitochondrial outer membrane import machinery (Kunkele et al. 1998; Su et al. 2022), a decline in TOMM20 may result in inefficient translocation of nuclear‐encoded TFAM and other proteins into the mitochondrial matrix (Garstka et al. 2003; Gensler et al. 2001). A sequence‐level contribution is also plausible. The two haplotypes differ within the D‐loop, the non‐coding region that contains the mitochondrial promoters and to which TFAM binds (Sathiaseelan et al. 2023; Nguyen et al. 2026), and variation in this region could reduce TFAM–mtDNA association independently of import. We did not assess TFAM occupancy at the D‐loop, and we regard these mechanisms as complementary rather than mutually exclusive. Given that TFAM is essential for mtDNA packaging and stabilization (Campbell et al. 2012), reduced mitochondrial TFAM abundance and TFAM–mtDNA binding may contribute to impaired mitochondrial genome maintenance, respiratory chain dysfunction, and bioenergetic failure, which is consistent with the decline in mitochondrial membrane potential observed at later age points. A comparable relationship has been described in humans. Across six admixed populations, mtDNA copy number declined as discordance between mitochondrial and nuclear genetic ancestry increased, an effect consistent in direction across haplogroups and attributed to suboptimal regulation of mtDNA replication when its components derive from different ancestries (Zaidi and Makova 2019). This parallels our observation of reduced mtDNA copy number accompanied by reduced binding of nuclear‐encoded TFAM to the mitochondrial genome, and suggests that the mitonuclear coordination we describe may be relevant to human populations of admixed ancestry. Together, our findings suggest a model in which the OKC‐HET^W^ haplotype may promote an early decline in mitochondrial protein import capacity, which would contribute to impaired TFAM‐mediated genome maintenance and ultimately promote ovarian aging and the emergence of numerous age‐related phenotypes. However, further studies are needed to elucidate the specific roles that other components of the mitochondrial protein import machinery such as other translocases of the outer (TOMM70, TOMM22, TOMM40) or inner membrane (TIMM23, TIMM22) may play (Bauer et al. 2000).

Importantly, the observed mitochondrial defects in OKC‐HET^W^ ovaries occurred independently of changes in overall mitochondrial mass or dynamics. Interestingly, the age‐dependent increase in FIS1 abundance in both cohorts indicates a shift toward mitochondrial fragmentation with advancing age, consistent with a previous study demonstrating that mitochondrial dynamics are shifted toward fission during aging of *Drosophila* ovarian germline stem cells (Amartuvshin et al. 2020). These observations indicate that mitochondrial quality, rather than quantity, distinguishes the two haplotypes.

Our longitudinal transcriptomic and proteomic analyses further supported these mechanistic findings and indicated that mitochondrial haplotype shapes the trajectory of ovarian aging. Interestingly, direct comparisons between OKC‐HET^B^ and OKC‐HET^W^ ovaries at each individual timepoint identified relatively few differentially expressed genes or proteins at early and mid‐reproductive ages, indicating that the early divergence in phenotypes we detected is not driven by widespread transcriptional reprogramming. With advancing age, differences due to haplotype became increasingly pronounced, particularly at the level of the proteome, where OKC‐HET^W^ ovaries demonstrated enrichment of mitochondrial dysfunction paired with coordinated suppression of pathways related to cell cycle progression, protein homeostasis, and cellular stress responses. Notably, the differentially abundant respiratory chain subunits were exclusively nuclear‐encoded, whereas the mtDNA‐encoded subunits detected in our dataset did not differ between haplotypes, indicating that the mitochondrial haplotype alters the abundance of nuclear‐encoded components of the respiratory chain rather than of the subunits it encodes directly. Because the expected nuclear allele dose is equivalent between cohorts by design, this places the mitochondrial genome upstream of a change in nuclear‐encoded protein abundance, consistent with altered coordination between the two genomes rather than an effect confined to either alone. This observation also argues against a technical explanation, since the mtDNA‐encoded subunits are the only proteins whose coding sequence differs between haplotypes and would therefore be the most susceptible to reference‐based quantification bias. In contrast, when comparing the rate of molecular change between young and advanced reproductive age, a striking bifurcation occurred. Compared to OKC‐HET^B^ counterparts, OKC‐HET^W^ ovaries underwent a far greater age‐dependent shift in transcriptome and proteome. This progression in OKC‐HET^W^ ovaries was marked by a robust activation of inflammatory and fibrotic signaling pathways, coupled with coordinated downregulation of processes related to protein synthesis, proteostasis, cell cycle regulation, and mitochondrial function. Upstream regulator analysis further strengthened this shift whereby pathways pertaining to ovarian function, cellular proliferation and mitochondrial activity were inhibited while those associated with inflammation and fibrosis were activated. These molecular signatures closely mimic the histological and functional phenotypes observed in OKC‐HET^W^ ovaries, including increased collagen deposition, macrophage infiltration, MNGC accumulation, and reduced ovulatory capacity.

Although our study provides experimental evidence linking mitochondrial genetic variation to the rate of ovarian aging, there are several limitations that should be noted. First, the OKC‐HET model was designed as a genetically heterogeneous population to better recapitulate the nuclear genetic diversity present in humans, rather than relying on a single inbred genetic background. Because both cohorts derive from reciprocal crosses of the same two F1 lines, the expected contribution of each founder strain to the autosomal genome is identical between cohorts, and nuclear variation is randomized with respect to haplotype rather than confounded with it. This constrains the genetic architectures consistent with our data. If the deleterious element resides in WKY nuclear DNA, with WKY mtDNA carrying a compensating variant, OKC‐HET^B^ animals would carry the nuclear liability without the mitochondrial compensation and would be the impaired cohort, which they are not. Although this design strengthens the translational impact of our model, it does not eliminate residual nuclear genetic variation as a potential contributor to the observed phenotypes. The reciprocal cross also does not balance the sex chromosomes: OKC‐HET^B^ and OKC‐HET^W^ females differ in the strain origin of the paternally inherited X chromosome, which is therefore not randomized with respect to mitochondrial haplotype. Given that several X‐linked genes influence ovarian function, a contribution from X‐linked variation cannot be formally excluded. Definitively distinguishing mitochondrial from mitonuclear architectures requires conplastic strains, in which a single haplotype is placed on a fixed isogenic nuclear background, and we regard this as the appropriate next experiment. We note that conplastic derivation requires approximately ten backcross generations to achieve near‐complete nuclear identity, which by conventional breeding takes more than 3 years (Houstek et al. 2012; Pravenec et al. 2007). Characterizing ovarian aging in inbred WKY females is a more tractable near‐term test of whether this haplotype confers accelerated ovarian aging on its own nuclear background, and one we are pursuing. Further genome‐wide characterizations of the nuclear genomes in these animals would also refine the interpretation of mitonuclear balance. Additionally, validation in other model systems and ultimately human cohorts will be required to define clinical relevance.

Second, the estrous cycle stage was not monitored during tissue collection. However, our previous work shows that ovarian transcriptome variation is primarily driven by age rather than cycle stage (Isola, Ocañas, et al. 2024). In addition, reproductive aging is associated with increasing estrous cycle irregularity, making strict cycle matching challenging in older animals. Importantly, animals of both haplotypes were collected under identical conditions at each age, so any variation in cycle stage is expected to be randomly distributed with respect to haplotype and would add variance rather than systematic bias. Third, the use of whole‐ovary approaches, including bulk transcriptomic, proteomic, and mitochondrial endpoint analyses, could mask cell‐type‐specific responses within the ovarian microenvironment. Appropriate future studies that use single‐cell approaches and/or compartment‐specific organoid studies will be required to understand how mitochondrial dysfunction and fibrotic remodeling occur at different cellular compartments. Fourth, the meiotic spindle analysis was performed on 30 oocytes per haplotype and, while the proportion of abnormal spindles was approximately two‐fold higher in OKC‐HET^W^ oocytes, this comparison was underpowered to establish statistical significance. Finally, although we pinpoint impaired TFAM‐mtDNA association and mitochondrial protein import as defining features of mt‐pathological states, the mechanism by which mitochondrial genetic variation disrupts nuclear‐encoded import pathways is not established. Despite these limitations, our findings provide experimental evidence that naturally occurring variation in mitochondrial haplotype influences the rate at which the ovary ages.

## 4 Conclusion

In conclusion, our study identifies mitochondrial genetic variation as a heritable modifier of the ovarian aging trajectory. We show that naturally occurring mitochondrial haplotype variation is associated with the rate of follicle depletion in a genetically heterogeneous population. Linking defective mitochondrial import and genome maintenance with a fibrotic, inflammatory microenvironment provides a framework for thinking about inter‐individual differences in reproductive lifespan. Although further studies are needed, our results suggest that mitochondrial targeted approaches may be beneficial strategies to help preserve ovarian health and improve female healthspan. Together, our studies position the mitochondrial genome not merely as a metabolic powerhouse but as a heritable factor acting in concert with the nuclear genome to shape the pace of ovarian aging and its systemic consequences.

## 5 Methods

### 5.1 Animals

Using four inbred rat strains (Brown Norway [BN], Fischer 344 [F344], Wistar Kyoto [WKY], and Lewis [LEW]) obtained from Charles River, OKC‐HET^B^ and OKC‐HET^W^ rats carrying distinct mitochondrial haplotypes were generated as previously reported (Sathiaseelan et al. 2023) and shown in Figure 1A. Briefly, BN/F344 and WKY/LEW F1 rats were first produced by crossing BN × F344 and WKY × LEW strains, respectively. Subsequent reciprocal breeding generated OKC‐HET^B^ and OKC‐HET^W^ rats, which carry different maternally inherited mitochondrial haplotypes on a shared diverse nuclear genetic background. Specifically, OKC‐HET^B^ rats harbor BN‐derived mtDNA, whereas OKC‐HET^W^ rats carry WKY‐derived mtDNA, which differ by 94 nucleotides (Sathiaseelan et al. 2023). Animals were housed and bred in specific pathogen‐free conditions at the Oklahoma City VA Medical Center and fed standard chow diet *ad libitum*. Reproductively young, mid, and advanced reproductive age female rats were used at 4, 9, and 14 months of age. At the designated ages, all animals were anesthetized (isoflurane) and euthanized via exsanguination using cardiac puncture before tissue collection. Blood was drawn into EDTA‐lined tubes, and plasma was isolated and stored at −80°C. Ovaries were harvested and either processed immediately for mitochondrial functional assays, flash frozen and stored at −80°C for molecular and biochemical analyses, or fixed in 4% paraformaldehyde (PFA), embedded, and serially sectioned for histological and immunofluorescence analyses. For all the experimental analyses, at least five females per haplotype and age group in each experiment were used unless specified otherwise. All procedures were in accordance with Institutional Animal Care and Use Committee at the Oklahoma City Veterans Affairs Health Care System.

### 5.2 Measurement of Anti‐Mullerian Hormone (AMH)

Plasma aliquots were sent to the University of Virginia Center for Research in Reproduction Ligand Assay and Analysis Core for measurement of AMH concentrations as described previously (Cinzori et al. 2023).

### 5.3 Histological Analyses

Ovaries (*n* = 5/haplotype/timepoint) were serially sectioned, and every twentieth section was stained with hematoxylin and eosin (H&E) for follicle quantification. Numbers of follicles at various stages of development (primordial, primary, secondary, tertiary) were counted based on criteria previously described (Wang et al. 2014; De La Torre et al. 2023). To account for sections not analyzed, counts of primordial, primary, and secondary follicles were multiplied by twenty, while tertiary follicle counts were adjusted by a factor of ten due to their larger size. Picrosirius red staining for collagen deposition and Sudan Black B staining for lipofuscin accumulation were performed on a randomly selected mid‐ovarian section from each animal and quantified as previously described (Isola, Ocañas, et al. 2024; Isola et al. 2025; Ansere et al. 2021).

### 5.4 Immunofluorescence Analyses

Immunofluorescence staining was performed on paraffin‐embedded ovarian sections as described earlier (Isola et al. 2025). Briefly, sections were deparaffinized, rehydrated, and subjected to antigen retrieval before blocking with serum to limit non‐specific binding. Slides were then incubated overnight at 4°C with the primary antibodies against CD45 (1:200; cat. no. ab10558, Abcam), F4/80 (1:1000; cat. no. ab300421, Abcam), and TOMM20 (1:500; cat. no. 42406T, Cell Signaling). Following primary antibody incubation, sections were washed and incubated with secondary goat anti‐rabbit IgG Alexa Fluor 488 antibody (1:1000; cat. no. 2338052, Jackson ImmunoResearch Laboratories) at room temperature for 1 h. Nuclei were counterstained with DAPI. Sudan Black B treatment was incorporated during the staining process to minimize tissue autofluorescence. Images were acquired using a confocal microscope (Zeiss LSM 880 with Airyscan) using green (F4/80 and TOMM20) and blue (DAPI) fluorescence channels at 10× magnification from three non‐overlapping ovarian regions per rat (*n* = 5/haplotype/timepoint). Mean fluorescence intensity was quantified using ImageJ after subtracting background from each image and averaging across three images per animal to obtain a representative value.

### 5.5 RNA Sequencing

Total RNA was isolated from one ovary per rat (*n* = 5/haplotype/timepoint) using the RNeasy Mini Kit (cat. no. 74104, Qiagen) according to the manufacturer's guidelines. cDNA libraries were prepared from 40 ng of total RNA after mRNA enrichment using the Watchmaker mRNA Library Prep kit (Watchmaker Genomics). Library size distribution was evaluated using High Sensitivity D1000 ScreenTape (Agilent Technologies), and library concentration quantified with the Qubit dsDNA HS Assay Kit on a Qubit 4 Fluorometer (Thermo Fisher Scientific). Indexed libraries were pooled at equimolar concentrations and sequenced on an Illumina NovaSeq X Plus platform for ~20 million reads per sample. The institution's high‐performance computing cluster was used to process raw sequencing reads (FASTQ files) using a custom bulk RNA‐seq pipeline. Read alignment was performed for the *Rattus norvegicus* reference genome (mRatBN7 with gene annotations from Ensembl release 111) using the STAR aligner (version 2.7.10b), and gene‐level counts were generated with featureCounts (version 1.6.3). DESeq2 was utilized in R (version 4.4.1) to carry out differential expression analysis. Group assignments were based on haplotype, condition, or combined haplotype‐condition labels as appropriate for each comparison. *p* values were adjusted for multiple testing (Benjamini‐Hochberg), and genes with an adjusted false discovery rate (FDR) < 0.05 were considered as significantly differentially expressed. Differentially expressed genes were further analyzed using Ingenuity Pathway Analysis (IPA) to identify enriched canonical pathways and upstream regulators. Heatmaps were generated using GraphPad Prism 9.0 software, and volcano plots were created in R using the ggplot2 package. RNA‐sequencing quality‐control metrics are provided in Figure S4.

### 5.6 Proteomic Analyses

Samples were processed according to previously published protocols with minor refinements (Isola et al. 2025; Newhardt et al. 2019). Briefly, homogenates from one ovary per rat (80 μg total protein per sample; *n* = 5/haplotype/timepoint) were denatured in SDS, supplemented with internal standard, and precipitated with acetone. Protein pellets were reconstituted in Laemmli buffer and subjected to SDS‐PAGE. Each gel lane was cut into small pieces, reduced, alkylated, and digested overnight using trypsin as a single sample. Peptides were extracted with acetonitrile‐containing buffer, dried under vacuum centrifugation, and reconstituted in 1% acetic acid for analysis. Data‐independent acquisition (DIA) was conducted on a Q Exactive Plus mass spectrometer using a 20 m/z isolation window over the range of *m*/*z* 350–950. The Orbitrap was operated at a resolution of 17,500 for DIA scans, with a full MS scan acquired at 70,000 resolution per cycle. Protein identification and quantification were performed by analyzing the DIA data with DIA‐NN against a *Rattus norvegicus* UniProt proteome database (Demichev et al. 2020). Proteins showing differences across experimental groups were then subjected to Ingenuity Pathway Analysis (IPA) to identify enriched canonical pathways and upstream regulators. Heatmaps and ranked horizontal bar plots were generated with GraphPad Prism 9.0 software, while volcano plots were created in R using the ggplot2 package.

### 5.7 Analysis of Mitochondrial Mass and Membrane Potential

Mitochondrial mass, membrane potential and ATP content were measured in freshly isolated ovarian tissue from 1 ovary per rat (*n* = 5/haplotype/timepoint). Single‐cell suspensions were made as previously described (Isola, Ocañas, et al. 2024). In brief, ovaries were enzymatically dissociated in DMEM (cat. no. D6429; Sigma) with collagenase (cat. no. C5138‐100MG, Sigma‐Aldrich) at 37°C with intermittent gentle pipetting to facilitate tissue digestion. After dissociation, cells were run through a 70 μm cell strainer, washed with DMEM and centrifuged to generate single‐cell suspension. Cells were resuspended in PBS, and suspension was divided into different fractions for JC‐1 staining, MitoTracker staining, and ATP quantification (Section 5.9). Mitochondrial membrane potential (ΔΨ_M_) was measured using the JC‐1 Mitochondrial Membrane Potential Assay Kit (cat. no. 1000972, Cayman) as directed by the manufacturer. For the JC‐1, cells were incubated with working solution of JC‐1 for 20 min at 37°C in the dark, washed twice and resuspended in assay buffer and analyzed by flow cytometry. ΔΨ_M_ was determined as the ratio of JC‐1 aggregates (high ΔΨ_M_; PE channel) to JC‐1 monomers (low ΔΨ_M_; FITC channel). Mitochondrial mass was assessed using MitoTracker Green FM (cat. no. 9074S, Cell Signaling Technology) at a final concentration of 1 μM, and fluorescence was analyzed by flow cytometry using the FITC channel. Mitochondrial content was measured as the geometric mean fluorescence intensity. All samples were analyzed on a BD FACSCelesta flow cytometer (BD Biosciences), and data were processed using FlowJo software (version 10.10.0).

### 5.8 Isolation of Mitoplasts and Measurement of Mitochondrial ETC Activity

Mitochondrial functions were measured using electron transport chain (ETC) enzyme activities. As previously described (Isola et al. 2025), mitoplasts were isolated from snap‐frozen whole ovaries. In brief, thawed tissues were homogenized in 400 μL of isolation buffer containing 1.0 mM EDTA and 25 mM MOPS pH 7.4 by motor‐driven Potter‐Elvehjem tissue grinder. The homogenate was spun down at 750 × g for 10 min at 4°C followed by another spin of the supernatant at 10,000 × g for 15 min. The resulting mitoplasts pellet was resuspended in 25 mM MOPS pH 7.4, and snap‐frozen immediately. To evaluate activity of the electron transport chain through complexes I‐III‐IV, NADH oxidase was measured. Frozen–thawed mitoplasts were diluted into 25 mM MOPS pH 7.4 to approximately 30 μg/mL and the rotenone‐sensitive rate of NADH oxidation was measured spectrophotometrically using an Agilent 8453 diode array UV–Vis spectrophotometer. Activity was quantified as the rate of NADH oxidation (340 nm, ε = 6200 M^−1^ cm^−1^) after adding 10 mM KCl and 150 μM NADH. Likewise, complex I activity was spectrophotometrically measured by the rate of NADH oxidation in the presence of 50 nM antimycin A and 100 μM ubiquinone‐1 following the addition of 150 μM NADH. Inhibition with 2.5 μM rotenone confirmed the specificity for complex I activity. The concentration of protein in each sample was measured using the Bradford method (using BSA as a standard).

### 5.9 ATP Quantification

ATP levels were measured using ATP Detection Assay Kit‐Luminescence (cat. no. 700410, Cayman Chemical) per the manufacturer's protocol. Briefly, fresh ovaries were processed immediately after collection to generate single‐cell suspensions (as described in Section 5.7). ATP measurements were performed on ice, and all samples were processed under identical experimental conditions to minimize ATP degradation. The single‐cell suspensions were lysed, and aliquots of cell lysates were used for ATP measurement. The luciferin‐luciferase reaction mixture was added in a 96‐well plate, and after mixing 10 μL of cell lysate, luminescence was read with a plate reader. ATP standards (0–10,000 fmol) were assayed in the same run to obtain a standard curve. Blank values were subtracted from all readings, and ATP concentrations were calculated by linear regression of log‐transformed standard curves. Protein concentration was determined by BCA assay, and ATP levels were normalized to protein content and expressed as fmol ATP per mg protein.

### 5.10 mtDNA Copy Number Assessment

Digital PCR was used to determine mitochondrial DNA (mtDNA) copy number using methods previously developed with minor adjustments (Masser et al. 2016). Briefly, total genomic DNA was isolated from one ovary per rat (*n* = 5/haplotype/timepoint) using the Qiagen AllPrep DNA/RNA Mini Kit following the manufacturer's protocol. Absolute quantification was done with the QuantStudio 3D Digital PCR System (Thermo Fisher Scientific) using a custom mtDNA TaqMan primer‐probe copy number assay (RnMtDNA_CCI1MPG, Thermo Fisher Scientific) (Masser et al. 2017). Reactions were prepared per the manufacturer's guidelines, loaded onto QuantStudio 3D digital PCR chips, and analyzed using the QuantStudio 3D AnalysisSuite software. mtDNA abundance was reported as the ratio of mtDNA copy number to nuclear DNA copy number, representing mtDNA copies per haploid genome equivalent.

### 5.11 TFAM Chromatin Immunoprecipitation (ChIP) Assay

ChIP was carried out using the ChromaFlash High‐Sensitivity ChIP Kit (EpigenTek, cat. no. P‐2027) in accordance with the manufacturer's instructions with slight variations. Briefly, 50 mg of rat ovarian tissue from 4‐month‐old female rats (*n* = 3/haplotype) were collected and minced before crosslinking in 1% formaldehyde for 15–20 min at room temperature. Crosslinking was quenched with glycine, and tissue was washed with cold PBS. Tissues were homogenized in lysis buffer containing protease inhibitors, and chromatin was extracted. Chromatin (2 μg per reaction) was incubated with anti‐TFAM antibody (0.8 μg per well) at room temperature as per kit specifications. RNA polymerase II and non‐immune IgG were used as positive and negative controls, respectively. After immunoprecipitation, crosslinks were reversed and DNA was purified using spin columns included in the kit. Purified ChIP DNA was quantified by digital PCR targeting mitochondrial genes (*mt‐Nd1* and *mt‐Co2*; Table S1), as described in Section 5.10. An equivalent aliquot of chromatin harvested before immunoprecipitation served as an input control. Fold enrichment was normalized as the ratio of TFAM‐associated mtDNA signal in the ChIP fraction versus in its corresponding input sample and is shown as relative TFAM binding to mtDNA. This normalization accounted for differences in total mtDNA abundance among haplotypes and allowed comparison of TFAM binding independent of baseline mitochondrial content.

### 5.12 Simple Western Analysis of Proteins Using Jess

Protein expression was measured by the Jess automated capillary Western system (ProteinSimple, Bio‐Techne) per manufacturer's instructions. For whole‐ovary protein analysis, rat ovarian tissue (*n* = 4/haplotype) was lysed in RIPA buffer containing protease and phosphatase inhibitors, and clarified by centrifugation at 10,000 × g for 10 min at 4°C. For subcellular fractionation, mitochondrial and cytoplasmic fractions were isolated from ovaries of 4‐month‐old OKC‐HET^B^ and OKC‐HET^W^ rats (*n* = 3/haplotype) using the Mitochondria Isolation Kit for Tissue (cat. no. 89801, Thermo Scientific) according to the manufacturer's instructions. Protein concentration was determined using the BCA assay. Protein lysates were diluted to 1 mg/mL in 0.1× sample buffer and then combined with 5× fluorescent master mix and denatured at 95°C for 5 min. Each capillary was loaded with a total of 3 μL per sample using a 12–230 kDa separation module. TFAM (cat. no. 22586‐1‐AP, Proteintech), TOMM20 (cat. no. 42406, Cell Signaling Technology), citrate synthase (cat. no. D7V88, Cell Signaling Technology), and GAPDH (cat. no. ab9485, Abcam) diluted 1:50 in antibody diluent were added to indicated wells followed by HRP‐conjugated secondary antibodies. Automatic protein separation, immunoprobing and chemiluminescent detection were carried out inside Jess system, and signals were analyzed using Compass software (ProteinSimple). For whole‐ovary lysates, TFAM abundance was normalized to GAPDH. For subcellular fractions, mitochondrial TFAM and TOMM20 abundance were normalized to citrate synthase (CS), while GAPDH served as a cytoplasmic marker to verify successful fractionation. Data are shown as normalized arbitrary units.

### 5.13 Assessment of Superovulation and Ovulation Rate

Superovulation was performed in 4‐month‐old female rats (*n* = 8/haplotype) following established methods (Lunn and Bell 1968). Briefly, animals were injected intraperitoneally with 12.5 IU equine chorionic gonadotropin (eCG), followed by a subsequent injection of 12.5 IU human chorionic gonadotropin (hCG) after 72 h. After 21 h of hCG administration, rats were euthanized and oviducts collected. Oviducts were placed in 40 μL drops of DMEM (cat. no. D6429, Sigma) supplemented with 5% BSA (cat. no. 130‐091‐376, Miltenyi Biotec). Cumulus‐oocyte complexes (COCs) were released by puncturing the ampulla under a stereomicroscope. COCs were transferred to DMEM containing 5% BSA and 100 U/mL hyaluronidase (cat. no. P5299, Abnova) and incubated at 37°C for 5–10 min to remove cumulus cells. Denuded oocytes were collected and counted. Ovulation rate was measured as the total number of ovulated oocytes recovered per animal.

### 5.14 Immunofluorescence Staining for Spindle Morphology

Denuded oocytes were retrieved following the superovulation protocol as described above. A total of 30 oocytes per haplotype were randomly selected from all recovered oocytes (obtained from 8 females per haplotype) for spindle morphology assessment according to previously reported methods (Suebthawinkul et al. 2022). Oocytes were fixed in 3.8% paraformaldehyde (containing 0.1% Triton X‐100) at 37°C for 20 min and subsequently washed with blocking buffer (1× PBS containing 0.01% Tween‐20, 0.02% sodium azide, and 0.3% BSA) followed by permeabilization in 1× PBS with 0.1% Triton X‐100, 0.02% sodium azide, and 0.3% BSA for 15 min at room temperature. After washing in blocking buffer, oocytes were incubated for 1 h at room temperature with anti‐α‐tubulin (11H10) rabbit monoclonal antibody, Alexa Fluor 488‐conjugated (1:100; Cell Signaling Technology, cat. no. 5063S) and Alexa Fluor 555 Phalloidin (1:50; Cell Signaling Technology, cat. no. 8953S) to visualize microtubules and F‐actin, respectively. Oocytes were washed three times in blocking buffer and mounted in Vectashield Antifade Mounting Medium with DAPI (Vector Laboratories) to visualize chromosome alignment. Images were acquired on a confocal microscope (Zeiss LSM 880 w/Airyscan) in the green (α‐tubulin), red (phalloidin), and blue (DAPI) channels at 63× magnification. Z‐stack optical sections were collected to assess meiotic spindle architecture. Spindles were considered normal if they displayed a bipolar structure and chromosomes were properly aligned at the metaphase plate.

### 5.15 Statistical Analyses

Data are expressed as mean ± SEM, unless otherwise stated. Statistical analyses were performed using GraphPad Prism 9.0 software unless specified otherwise. For comparisons between two haplotypes at one time point, unpaired two‐tailed Student's *t*‐tests were performed. For experiments involving both haplotype and age as variables, two‐way ANOVA was performed followed by Tukey's multiple comparisons test when appropriate. A value of *p* < 0.05 was considered statistically significant for non‐omics analyses. For RNA sequencing, differential gene expression was analyzed in R using DESeq2 with the Wald test. *p* values were adjusted for multiple testing using the Benjamini‐Hochberg method, and genes with an adjusted false discovery rate (FDR) < 0.05 and |log_2_FC| > 0.3 were considered significantly differentially expressed. For proteomic analyses, differential protein abundance was assessed using two‐way ANOVA with haplotype and age as factors, followed by Tukey's post hoc testing for between‐ and within‐haplotype age comparisons. Proteins with *p* < 0.05 and |log_2_FC| > 0.3 were considered significantly altered. For pathway analyses (RNA‐seq and proteomics), differentially expressed genes or proteins were imported into Ingenuity Pathway Analysis (IPA), and enrichment *p* values generated within IPA were considered significant at *p* < 0.05.

## Author Contributions

S.B., A.R., and M.B.S. conceived the project and designed the experiments. S.B. performed the experiments with contributions from H.V.M.N, A.C., S.M., M.T.K., K.M.H., W.M.F., S.R.O., F.E.D., T.L.L. Jr., and S.B. created figures and performed statistical analyses. S.B. and M.B.S. wrote the manuscript and all authors edited and approved the final version.

## Funding

This work was supported by grants from the National Institutes of Health (R33 AG072137 to M.B.S and A.R.; R01 AG069742 to M.B.S.; R01 AG099844 to M.B.S. and S.R.O.; P30 AG050911 to M.T.K and W.M.F.; P20 GM103447 to M.T.K.; P20 GM139763 to K.M.H.; R35 GM137921 to T.L.L Jr.; R01 HD105752 to F.E.D.), the Global Consortium for Reproductive Longevity and Equality (GCRLE‐4501 to M.B.S.; GCRLE‐0523 to S.R.O.) and the Department of Veterans Affairs (1IK6BX005238 and I01BX004538 to A.R; IK6BX006033 to W.M.F.). The funders had no role in study design, data collection and analysis, decision to publish, or preparation of the manuscript. The authors would like to thank the OMRF Clinical Genomics Center, Imaging Core Facility, and Flow Cytometry and Cell Sorting Core Facility for assistance with assay optimization and instrument setup, and the OMRF Center for Biomedical Data Sciences for data processing and analysis support.

## Conflicts of Interest

The authors declare no conflicts of interest.

## Supporting information


**Figure S1:** Mitochondrial haplotype influences the accumulation of immune cells. (A) Representative immunofluorescence images of ovarian CD45 positivity (red) and nuclei (blue), and (B) Quantification of ovarian leukocyte abundance expressed as mean CD45 fluorescence intensity measured within regions of interest of constant area from OKC‐HET^B^ and OKC‐HET^W^ at 4, 9, and 14 months of age. All data are presented as mean ± SEM and analyzed by two‐way ANOVA with Tukey post hoc test. *n* = 5/haplotype/timepoint/assay; **p < 0.01; ***p < 0.001.
**Figure S2:** TFAM expression and age‐associated TOMM20 abundance in OKC‐HET^B^ and OKC‐HET^W^ ovaries. (A) Quantification of TFAM transcript abundance from normalized ovarian bulk RNA‐sequencing data, (B) Quantification of TFAM protein abundance by immunoblotting with representative immunoblots in ovaries from OKC‐HET^B^ and OKC‐HET^W^ rats at 4 months of age, (C) Representative immunofluorescence images of TOMM20 positivity (green) and nuclei (blue) (scale bar = 100 μm), and (D) Quantification of TOMM20 abundance expressed as mean CD45 fluorescence intensity measured within regions of interest of constant area in ovaries from OKC‐HET^B^ and OKC‐HET^W^ rats at 4, 9, and 14 months of age. All data are presented as mean ± SEM and analyzed using *t*‐tests (panel A, B) or two‐way ANOVA with Tukey post hoc testing (panel C, D), as appropriate. *n* = 4–5/haplotype/timepoint/assay; **p < 0.01; ***p < 0.001.
**Figure S3:** Ovarian mitochondrial mass and regulators of mitochondrial dynamics are unaffected by mitochondrial haplotype. (A) Flow cytometry‐based quantification of mitochondrial mass in single‐cell ovarian suspensions using MitoTracker staining, (B) Relative abundance of the mitochondrial fission protein FIS1, (C) Relative abundance of the mitochondrial fusion protein MFN1, and (D) Schematic summary illustrating age‐associated increases in mitochondrial fission (FIS1↑) without corresponding changes in mitochondrial fusion (MFN1↔) from OKC‐HET^B^ and OKC‐HET^W^ ovaries at 4, 9, and 14 months of age. All data are presented as mean ± SEM and analyzed by two‐way ANOVA with Tukey post hoc test. *n* = 5/haplotype/timepoint/assay; *p < 0.05; **p < 0.01; ***p < 0.001.
**Figure S4:** RNA‐sequencing quality‐control and sensitivity analyses. (A) Euclidean distance between the 4‐ and 14‐month transcriptomic centroids of OKC‐HET^B^ and OKC‐HET^W^ ovaries calculated from variance‐stabilized transformed (VST) gene‐expression values using all samples (“All”) and sequential leave‐one‐out analysis of individual 14‐month biological replicates, (B) DESeq2 dispersion estimates for the 14‐ versus 4‐month comparisons in OKC‐HET^B^ and OKC‐HET^W^ ovaries. Gene‐wise estimates (black), fitted dispersion trends (red), and final dispersion estimates (blue) are shown, (C) RNA Integrity Number (RIN) values for RNA‐sequencing samples across age and haplotype (*n* = 5/haplotype/age), (D) FastQC mean per‐base Phred quality scores across RNA‐sequencing libraries, and (E) Sequencing depth and uniquely mapped read rates for OKC‐HET^B^ and OKC‐HET^W^ RNA‐sequencing libraries. Values are presented as mean ± SD (*n* = 15/haplotype).
**Table S1:** Oligonucleotide primers used for ChIP‐based quantification of TFAM‐mtDNA binding.

## Data Availability

The datasets generated through this work are available in a publicly accessible repository. The RNA‐sequencing datasets generated in this study have been deposited in the NCBI Gene Expression Omnibus (GEO) under accession number GSE328056. Raw proteomics data have been deposited in Zenodo under DOI 10.5281/zenodo.21282707. Differential gene and protein expression results are provided as Supporting Information Files S1.
