# Supplementary material for: Mitochondrial Haplotype Shapes the Trajectory of Ovarian Aging in Genetically Heterogeneous Rats
Source: Aging Cell. 2026 Sep 10;25(9):e70710. doi: 10.1111/acel.70710 (PMC13562602; doi:10.1111/acel.70710)
Supplement: Supplementary file 1 [file ACEL-25-e70710-s001.docx]

**Main Title:** Mitochondrial haplotype shapes the trajectory of ovarian aging in genetically heterogeneous rats

**Running Title:** Mitochondrial haplotype in ovarian aging

Subhasri Biswas^1^, Hoang V.M. Nguyen^2^, Aubrey Converse^3^, Satoshi Matsuzaki^1^, Michael T. Kinter^1^, Kenneth M. Humphries^1^, Willard M. Freeman^4,5^, Sarah R. Ocañas^4,5^, Arlan Richardson^2,5^, Francesca E. Duncan^3^, Tommy L. Lewis Jr^1^, Michael B. Stout^1,5^*

**SUPPLEMENTARY INFORMATION (FIGURES & TABLE)**

**Figure S1. Mitochondrial haplotype influences the accumulation of immune cells.** (A) Representative immunofluorescence images of ovarian CD45 positivity (red) and nuclei (blue), and (B) Quantification of ovarian leukocyte abundance expressed as mean CD45 fluorescence intensity measured within regions of interest of constant area from OKC-HET^B^ and OKC-HET^W^ at 4, 9, and 14 months of age. All data are presented as mean ± SEM and analyzed by two-way ANOVA with Tukey post-hoc test. n=5/haplotype/timepoint/assay; **p<0.01; ***p<0.001.

**Figure S2. TFAM expression and age-associated TOMM20 abundance in OKC-HET^B^ and OKC-HET^W^ ovaries.** (A) Quantification of TFAM transcript abundance from normalized ovarian bulk RNA-sequencing data, (B) Quantification of TFAM protein abundance by immunoblotting with representative immunoblots in ovaries from OKC-HET^B^ and OKC-HET^W^ rats at 4 months of age, (C) Representative immunofluorescence images of TOMM20 positivity (green) and nuclei (blue) (scale bar = 100 μm), and (D) Quantification of TOMM20 abundance expressed as mean CD45 fluorescence intensity measured within regions of interest of constant area in ovaries from OKC-HET^B^ and OKC-HET^W^ rats at 4, 9, and 14 months of age. All data are presented as mean ± SEM and analyzed using t-tests (panel A-B) or two-way ANOVA with Tukey post-hoc testing (panel C-D), as appropriate. n=4-5/haplotype/timepoint/assay; **p<0.01; ***p<0.001.

**Figure S3. Ovarian mitochondrial mass and regulators of mitochondrial dynamics are unaffected by mitochondrial haplotype.** (A) Flow cytometry-based quantification of mitochondrial mass in single-cell ovarian suspensions using MitoTracker staining, (B) Relative abundance of the mitochondrial fission protein FIS1, (C) Relative abundance of the mitochondrial fusion protein MFN1, and (D) Schematic summary illustrating age-associated increases in mitochondrial fission (FIS1↑) without corresponding changes in mitochondrial fusion (MFN1↔) from OKC-HET^B^ and OKC-HET^W^ ovaries at 4, 9, and 14 months of age. All data are presented as mean ± SEM and analyzed by two-way ANOVA with Tukey post-hoc test. n=5/haplotype/timepoint/assay; *p<0.05; **p<0.01; ***p<0.001.

**Figure S4. RNA-sequencing quality-control and sensitivity analyses.** (A) Euclidean distance between the 4- and 14-month transcriptomic centroids of OKC-HET^B^ and OKC-HET^W^ ovaries calculated from variance-stabilized transformed (VST) gene-expression values using all samples (“All”) and sequential leave-one-out analysis of individual 14-month biological replicates, (B) DESeq2 dispersion estimates for the 14- versus 4-month comparisons in OKC-HET^B^ and OKC-HET^W^ ovaries. Gene-wise estimates (black), fitted dispersion trends (red), and final dispersion estimates (blue) are shown, (C) RNA Integrity Number (RIN) values for RNA-sequencing samples across age and haplotype (n = 5/haplotype/age), (D) FastQC mean per-base Phred quality scores across RNA-sequencing libraries, and (E) Sequencing depth and uniquely mapped read rates for OKC-HET^B^ and OKC-HET^W^ RNA-sequencing libraries. Values are presented as mean ± SD (n = 15/haplotype).

**Table S1. Oligonucleotide primers used for ChIP-based quantification of TFAM-mtDNA binding**

| Gene | Sequence (5′-3′) |
| --- | --- |
| *mt-Nd1* | Probe: 5’-/56-FAM/ACCCTAGCA/ZEN/GAAACAAATCGAGCTCC/3IABkFQ/-3’  Primer 1: 5’-CGACGTTAAAGCCTGAGACTAA-3’  Primer 2: 5’-CGCCTGACCAATAGCCATAA-3’ |
| *mt-Co2* | Probe: 5’-/56-FAM/CTGGAGGTG/ZEN/GAGACCCAATCCTTT/3IABkFQ/-3’  Primer 1: 5’-GGTGGCCGAAGAATCAGAATAG-3’  Primer 2: 5’-GCCAGTATTAGCAGCAGGTATC-3’ |
